# Temporal Changes of Metabolic Indicators and Quality of Life by a Two-Day Patient Education Program for Metabolic Syndrome Patients

**DOI:** 10.3390/ijerph19063351

**Published:** 2022-03-12

**Authors:** Jeong Suk Jeon, Sang Yeoup Lee, Soon Cheol Ahn, Yun Jin Kim, Jeong Gyu Lee, Yu Hyeon Yi

**Affiliations:** 1 Family Medicine Clinic, Bongseng Memorial Hospital, 48775 Busan, Korea; dalkiiis@hanmail.net; 2 Family Medicine Clinic, and Biomedical Research Institute, Pusan National University Yangsan Hospital, 50612 Yangsan, Korea; 3 Department of Medical Education, Pusan National University School of Medicine, 50612 Yangsan, Korea; 4 Department of Microbiology & Immunology, Pusan National University School of Medicine, 50612 Yangsan, Korea; ahnsc@pusan.ac.kr; 5 Department of Family Medicine, Pusan National University School of Medicine, 50612 Yangsan, Korea; yujkim@pusan.ac.kr (Y.J.K.); eltidine@hanmail.net (J.G.L.); eeugus@hanmail.net (Y.H.Y.)

**Keywords:** education, health, metabolic syndrome, nutrition, patient, physical activity

## Abstract

Metabolic syndrome (MetS) is a disease with a high prevalence that threatens the health of modern people. Patient education is essential to control MetS. This prospective study aimed to evaluate 6-month changes in health indicators following a two-day education program for patients with MetS aged 45 or older. Education about MetS, lifestyle modification, nutrition, and physical activity was provided. At 3 and 6 months after the program, participants visited for follow-up. Twenty-two patients completed the 6-month study. Waist circumference was reduced, and life quality and depression index improved in 3 and 6 months compared to pre-education. Blood pressure decreased, and anxiety index improved at three months. Nutritional knowledge was well maintained for 3 and 6 months. High-density lipoprotein-cholesterol levels increased at six months. Three out of twenty-two patients did not satisfy MetS criteria at the end of the study due to improved indicators. A two-day multidisciplinary education program positively affected health indicators in MetS patients. Participation in the program also help with life satisfaction and positive emotional condition. However, some indicators improved in 3 months, but the effect disappeared 6 months after the program.

## 1. Introduction

Metabolic syndrome (MetS) is a major risk factor for cardio- and cerebrovascular disease. Chronic diseases, such as diabetes, hypertension, dyslipidemia, and obesity, which are components of MetS, are associated with less healthy lifestyle habits [1,2]. Medication prescribed to control these diseases needs to be taken over a long duration; therefore, the treatment effect is highly related to a patient’s adherence to the medication. Moreover, to improve MetS, it is essential to improve a patient’s health behavior concerning medication. Therefore, to improve treatment adherence and the lifestyle habits of patients, a high level of understanding of their underlying disease is required.

An illness can be better controlled when a patient is made aware of disease-related knowledge, such as disease characteristics, disease criteria, treatment methods, and self-regulation [3]. Patients generally wish to understand their disease and seek information. However, patient education has not always been entirely achievable due to time and space restrictions. In addition, MetS and depression are closely related [4], and a lower quality of life for patients with MetS has been reported [5]. Patients with MetS require a psychological approach to improve depression and quality of life as well as education when treating this syndrome. We considered that, through undertaking an education camp for patients with a similar disease to form a supportive peer group, knowledge transfer to the patients through education could occur, and improvements in quality of life could be expected as patients collectively shared challenges and experiences and formed an emotional support network. Especially, the multidisciplinary education movement has already become an inevitable trend for improving health [6]. Teamwork is essential when taking care of complex and chronic diseases. From the long-term perspective, multidisciplinary education also improves the quality of care, patient outcomes, and patient care costs [7]. However, despite the need for effective and high-quality multidisciplinary education through an interdisciplinary team in the field of health care to improve the health status of MetS patients, research on the effectiveness of multidisciplinary education in MetS is still lacking. Therefore, this study aimed to assess whether a two-day multidisciplinary education program could initiate a healthy lifestyle, improve health indicators through lifestyle change, increase awareness of the disease, enhance sound mental health, or improve quality of life for patients with MetS.

## 2. Materials and Methods

### 2.1. Study Design

A two-day patient education camp was planned by Memorandum of Understanding between Yangsan City and Busan National University Yangsan Hospital. This prospective study analyzed the effectiveness of the camp by conducting a follow-up survey at 3 and 6 months after the two-day camp. A public health center in Yangsan hosted this camp, and the city of Yangsan covered all expenses. Physicians, nurses, nutritionists, and social workers belonged to Pusan National University Yangsan Hospital, while sports experts belonged to the department of physical education at Kyungnam University. All staff volunteered.

The study (NCT04867239) was approved by the Institutional Review Board at Pusan National University Yangsan Hospital (IRB No. 05–2014–039). The participants were informed concerning the study aims, and their written consent was obtained prior to the administration of the study. All methods were performed in accordance with the relevant guidelines and regulations set out in the Declaration of Helsinki. Participation was voluntary and anonymous, and any participant could withdraw from completing the questionnaire. Anonymity and data confidentiality were ensured.

### 2.2. Participants

A prospective study was conducted for six months in Pusan National University Yangsan Hospital and Yangsan Public Health Center, South Korea. We handed out leaflets and hung banners saying we would recruit camp participants with obesity, hyperlipidemia, diabetes, or high blood pressure. Of those screened, only people who met the MetS criteria joined the camp. Twenty-nine patients with MetS aged ≥ 45 years residing in Yangsan, South Korea, participated in a two-day multidisciplinary education camp in May 2014. MetS was diagnosed using the harmonizing definition (2009) proposed by the American Heart Association—International Diabetes Federation. Central obesity was defined according to the suggested definition of the Korean Society for the Study of Obesity [8]. During the follow-up period, 5 participants dropped out because they did not visit the health center, and 2 participants were dropped out due to not completing a blood test at six months. Finally, we analyzed the results of 22 participants. There were no differences in baseline variables between the 22 follow-up completers and the seven non-completers.

### 2.3. A Two-Day Multidisciplinary Education Program

**Figure 1:**
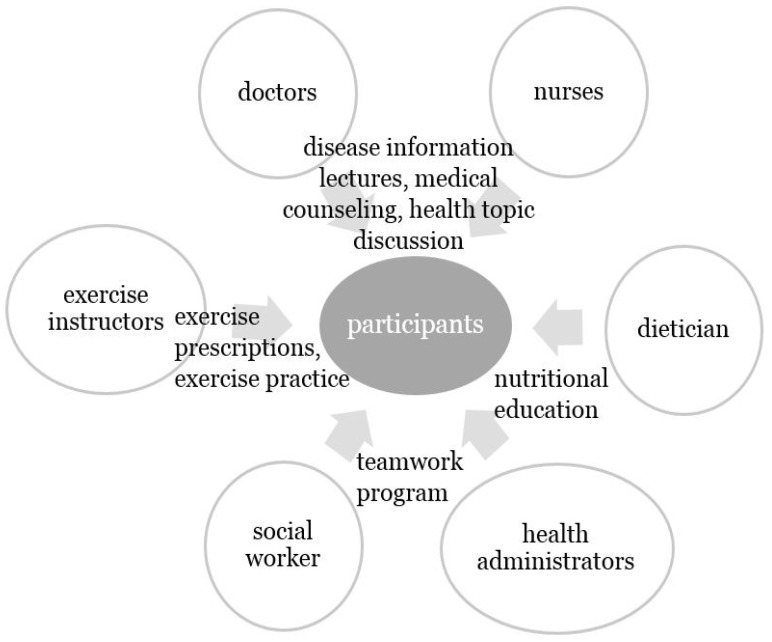
A two-day multidisciplinary education camp program.

A multidisciplinary professional team comprised of physicians, nurses, exercise instructors, nutritionists, social workers, and health administrators attended the program for the management and prevention of MetS (Figure 1). On the first day of camp, participants indicated on a survey sheet whether they were currently diagnosed with hypertension, diabetes, hyperlipidemia, and other diseases. Then, anthropometric and BP measurements and over 8-hour fasting blood tests were undertaken. Physicians and nurses conducted lectures for 30 minutes and 40 minutes each on chronic diseases, such as hypertension, hyperlipidemia, diabetes, and MetS. The contents included disease information and healthy lifestyle habits for disease control. Following this, physicians provided individual participants with further details covering health-related topics. A nutritionist provided participants with nutritional education and information concerning both calorie count and nutrition and advised participants on recommended amounts of food for each meal. Exercise instructors gave lectures and prescriptions on the appropriate intensity, frequency, and amount of exercise. Furthermore, a cognitive enhancement fitness program undertaken with an exercise instructor was conducted for 120 minutes to simultaneously stimulate motor nerves and the central nervous system to enhance integrated function. The participants then gathered in small groups of 10 and participated in an emotional support group program with social workers. At the end of the first day, exercise instructors performed physiotherapy. On the second day, physicians conducted individual consultations with participants on the management of MetS based on the previous day’s results. As instructed on the first day, the participants selected their nutritional formula. All participants (*n* = 29) completed BP measurements, blood tests, and questionnaires at the start of the camp, and then, anthropometric and BP measurements, blood tests, and questionnaires were followed up at 3- and 6-months post-education in a public health center. 

### 2.4. Measurements

Height was measured using an automatic stadiometer, with participants placed in a standing position wearing light attire and no shoes. Bodyweight and body fat percentage were measured using a body composition analyzer (Olympia 3.3, Jawon Medical, KyungSan, Korea). Body mass index (BMI) was calculated as weight (kg) divided by height squared (m^2^). Waist circumference was measured midway between the lowest rib and the top of the iliac crest at the end of expiration using non-elastic tape. A mercury-free sphygmomanometer was used to measure BP, with participants placed in a seated position after a 10 min rest period. BP levels were recorded pre-education and at 3 and 6 months after education. Blood tests were taken after the participants had fasted for 8 h at the start of the camp and six months after education. HbA1c levels were tested with ion-exchange chromatography using an automatic biochemical analyzer, Variant II (Bio-Rad, Hercules, CA, USA). Total cholesterol, triglyceride, and high-density lipoprotein (HDL) cholesterol levels were measured directly through an enzymatic method using a point-of-care analyzer (Cholestech LDX, Green Medical, Seoul, Korea). Low-density lipoprotein (LDL) cholesterol concentrations were calculated using the Friedewald formula.

### 2.5. Questionnaires

Participants were requested to respond to six kinds of self-administered questionnaires to evaluate the quality of life, stress, depression, anxiety, physical activity, and nutritional knowledge. Quality of life was assessed using the Korean EQ-5D (EuroQol 5-dimension scale) [9]. Anxiety, depression, and stress were evaluated using the Beck anxiety inventory (BAI), the Beck depression inventory (BDI), and the Korean version of the Brief Encounter Psychosocial Instrument (BEPSI-K), respectively [10,11,12]. Physical activity was assessed using the International Physical Activity Questionnaire (IPAQ), and the continuous-scoring method was used to calculate and add up all the activities over one week [13]. Nutritional knowledge was evaluated using the nutritional knowledge questionnaire (NKQ). The research team developed this knowledge assessment to assess nutrition knowledge for the management of MetS based on nutrition education conducted during camp on the topic of MetS-related nutrients. All six questionnaires were completed at the start of the camp and again 3 and 6 months after education. 

### 2.6. Statistical Analysis

The mean (standard deviation) or median (interquartile range) for the numerical data and the frequency (percent) for each categorical data are presented. Data, except age, were not normally distributed when checked using the Shapiro-Wilk test. We used Wilcoxon signed-rank test for non-parametric variables. *p*-Values of < 0.05 were deemed statistically significant. All statistical tests were two-sided. SPSS version 22.0 (SPSS Statistics for Windows Version 22.0, Armonk, NY, USA, IBM Corp) was employed for the analysis.

## 3. Results

### 3.1. Baseline Characteristics of the Study Participants

**Figure 2:**
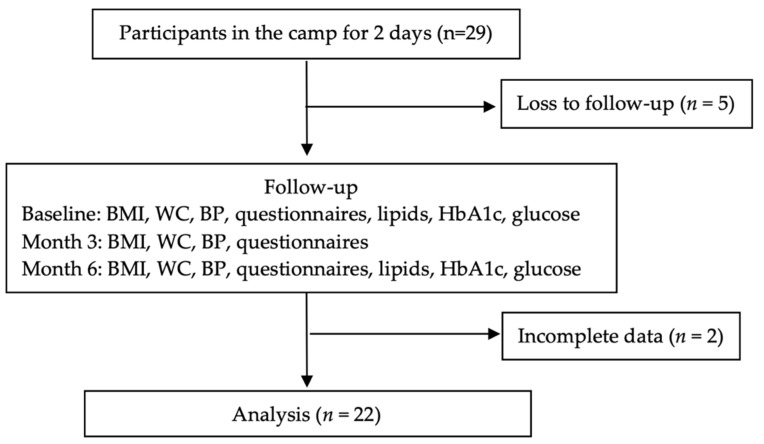
Flow diagram of study. BMI, body mass index; WC, waist circumference; BP, blood pressure.

Of the 29 camp participants, 22 participants who had completed the follow-up visits were included in the analysis (Figure 2). The mean age was 62.0 (± 6.1) years, and 20 (90.9%) participants were female. Sixteen participants (72.7%) had been diagnosed with hypertension, four (18.2%) had been diagnosed with diabetes, and sixteen (72.7%) had been diagnosed with hyperlipidemia by self-report (Table 1).

### 3.2. Changes in Anthropometric and BP Measurement

Bodyweight, BMI, and body fat percentage did not differ pre- and post-education attendance. Waist circumference measurements showed a significant decrease at 3 months (*p* = 0.001) and 6 months (*p* = 0.004). BP levels decreased compared to pre-education BP levels at 3 months (systolic BP, *p* = 0.015; diastolic BP, *p* = 0.012) significantly (Table 2 and Table 3).

### 3.3. Changes in Questionnaire Scores

The EQ-5D scores significantly increased both at 3 months (*p* = 0.029) and 6 months (*p* = 0.034). The EQ visual analog scale also significantly increased both at 3 months (*p* = 0.031) and 6 months (*p* = 0.015). The BDI scores decreased significantly at 3 months (*p* = 0.009) and 6 months (*p* = 0.012). The BAI scores decreased at 3 months (*p* = 0.025) compared to the pre-education scores. The NKQ scores increased significantly at 3 months (*p* = 0.005) and 6 months (*p* = 0.001). The BEPSI-K scores increased at post-education compared to pre-education, but this was not statistically significant. The physical activity score did not differ between pre-and post-education (Table 2 and Table 3).

### 3.4. Changes in Laboratory Tests Results

At six months, HDL cholesterol levels showed a more statistically significant increase (*p* = 0.007) than pre-education HDL cholesterol levels. LDL cholesterol levels at six months also decreased but were not significant. However, fasting blood sugar (*p* < 0.01), HbA1c (*p* < 0.01), triglyceride (*p* < 0.01), and total cholesterol (*p* < 0.05) values recorded at six months increased compared to pre-education values (Table 2 and Table 3).

### 3.5. Improvement of Metabolic Syndrome

At the start of the camp, all 22 participants satisfied the criteria of MetS. At six months, three of them (13.7%) had improved some components of the MetS criteria, including a decrease in the waist circumference, increased HDL cholesterol, and decreased triglyceride. As a result, they were no longer MetS (Table 2 and Table 3).

## 4. Discussion

This study found that a multidisciplinary education camp for two days had an effect that lasted for six months in patients with MetS. The impact of education was directly effective on indicators of MetS, such as reduced waist circumference, decreased BP, and increased HDL cholesterol. It was also confirmed that it had beneficial effects on emotional conditions, such as increased satisfaction with life and reduced depression and anxiety. Regarding nutritional knowledge, the degree of knowledge was maintained at a higher level at post-education than pre-education for six months, which suggests that the acquired knowledge is likely to be retained if the patient education program is of a high standard.

BP and anxiety index showed positive results only at three months but not at six months after education. We provided patient education only at the camp and did not offer any further education during the follow-up period. It is necessary to ensure that motivation is strengthened and sustained through additional education to maintain healthy lifestyle changes, such as continuous physical training and education sessions [14]. We recommend providing motivation and goals to patients every 3 months based on our results.

Although our multidisciplinary education camp was only for two days, it effectively improved depression, anxiety, and quality of life, as similar types of patients shared their experiences and received the same education together. The psychological effects appeared to last long after the camp had ended. A previous study showed that patients with diabetes and high stress levels who collectively shared their experiences reduced their burden of living with diabetes regardless of glycemic control [15]. These results suggest that experiential learning at an education camp can be an effective way to improve depression and increase life satisfaction for patients with MetS.

Previously, a few studies reported the positive result of camp-based health programs. In a study concerning a weight-loss education camp conducted over ten weeks for obese children, indicators of MetS, including weight loss, improved after education compared with before education [16]. In another study involving a 3-day education camp for 30 patients with diabetes, fasting plasma glucose reduction and an increase in self-esteem were reported at the six months after education compared with pre-education measurements [17]. In a study of a 5-day diabetes education camp, participants were followed up for a further six months. At three months after education, HbA1c levels were decreased significantly but had increased again at six months after education although the psychological well-being and knowledge those patients had gained during the camp persisted for up to 6 months [18]. This study is similar to our results in that the effectiveness of education was maintained by only about three months on some indicators. In our study, after six months, the waist circumference decreased, and the depression and quality of life indicators improved. Nutritional knowledge was also maintained after six months. On the other hand, after six months, median values of disease markers except for HDL cholesterol deteriorated. Blood pressure decreased at three months but returned to the initial levels at six months. In interviews by phone with several participants after six months, some of them misunderstood their education at this camp. They thought they could no longer take their medication if they improved their diet, exercise, and lifestyle and eventually stopped taking medicines arbitrarily without consulting their doctor. Perhaps such misunderstandings contributed, at least in part, to the worsening of disease markers after six months. In another study of a 7-day diabetes camp for children with type 1 diabetes, when education and motivation were provided for each visit of a 12-month follow-up, data showed that HbA1c levels reduced significantly at the 12-month mark [19]. Therefore, continuous education and incentive will be necessary for maintaining lifestyle modifications, encouraging physical activity, and correctly understanding how the disease is managed. 

The present study results and other previous studies [14,15,16,17,18,19] suggest that camp-based health programs are valuable means of patient education. Our camp participants enrolled voluntarily in the program and may have been more motivated and active than other general patient groups. Participants at the camp were of similar ages and suffered from the same medical problems. They could share experiences about their illness and difficulties. Previous studies reported that sharing experiences could positively affect anxiety and depression [15]. Educating and counseling concerning diseases in a more open atmosphere external to a clinic has been reported to help maintain knowledge for a more extended period [20].

The limitation of our study was the lack of a control group and a small number of enrolled participants, which made results difficult to generalize to a larger population or similar populations. Second, this could be a selection bias because our campers, who voluntarily enrolled in the program, were motivated and willing to practice the education they received through this program. Regardless of limitations, this study suggests a role for a multidisciplinary education camp program in managing MetS. 

## 5. Conclusions

A two-day multidisciplinary education camp for patients with MetS improved MetS indicators, such as BP, waist circumference, and HDL cholesterol. The participation of the camp-type education program also helps with life satisfaction and positive emotional condition. To consistently reduce patients’ disease indices, ongoing motivation is necessary through periodic group or individual education every three months.

## Figures and Tables

**Table 1:** Pre-education data of the study population (*n* = 22).

Variables		Baseline
Age (years)		62.1 ± 6.0
Women		20 (90.9)
Comorbidities	Hypertension	16 (72.7)
	Diabetes	4 (18.2)
	Dyslipidemia	16 (72.7)

Values are expressed as the means ± standard deviations (SD) or number (%).

**Table 2:** Results of pre- and post-camp data.

Variables		Pre-Camp	Post-Camp	
		Baseline	3 Months	6 Months
Health indices	Weight (kg)	57.9 (54.3–67.9)	57.9 (53.7–67.6)	57.9 (54.4–68.7)
	Body Mass Index (kg/m^2^)	24.6 (22.4–26.9)	24.1 (22.1–26.7)	24.0 (21.9–26.8)
	Waist Circumference (cm)	89.0 (85.3–91.8)	85.0 (79.3–89.9) **	86.0 (78.3–91.5) **
	Body Fat (%)	31.0 (29.8–33.5)	30.0 (29.7–33.4)	30.5 (29.5–34.2)
	Systolic BP (mmHg)	131.0 (126.5–140.8)	130.0 (118.5–134.8) *	137.0 (131.0–144.0)
	Diastolic BP (mmHg)	76.0 (70.3–80.0)	73.0 (66.0–77.5) *	82.0 (73.0–87.0)
Quality of life	EuroQol 5-dimension Scale	0.96 (0.87–0.96)	1.00 (0.92–1.00) *	0.96 (0.96–1.00) *
	EuroQol 5 VAS	70.0 (50.0–80.0)	70.0 (66.3–90.0) *	80.0 (70.0–88.8) *
	BEPSI-K	20.0 (15.5–22.0)	21.0 (19.0–23.0)	21.0 (20.0–24.0)
Emotional conditions	Beck Depression Inventory	8.0 (3.5–20.8)	6.0 (2.0–10.0) **	5.0 (2.3.0–10.8) *
	Beck Anxiety Inventory	12.0 (4.0–19.8)	6.0 (2.0–10.0) *	6.0 (2.3–10.8)
Physical activity	International Physical Activity Questionnaire (METs)	3150.0 (1811.8–4047.0)	2586.0 (1037.3–3726.8)	3132.0 (1707.0–4150.5)
Disease knowledge	Nutritional Knowledge Questionnaire	8.0 (7.0–9.8)	9.0 (8.0–10.8) **	9.0 (9.0–11.0) **
Disease markers	Fasting Glucose (mg/dL)	90.0 (84.3–97.8)		110.0 (93.8–131.0) **
	Total Cholesterol (mg/dL)	162.0 (142.0–183.0)		171.0 (156.0–193.5) *
	HDL Cholesterol (mg/dL)	45.0 (36.8–53.0)		49.0 (40.0–60.5) **
	Triglyceride (mg/dL)	108.0 (68.0–137.0)		136.0 (102.3–229.8) **
	LDL Cholesterol (mg/dL)	93.0 (69.0–115.0)		88.5 (68.0–108.0)
	HbA1c (%)	5.4 (5.3–5.6)		5.6 (5.5–5.8) **

Results are presented as median (interquartile range). BP, blood pressure; VAS, visual analogue scale; BEPSI-K, Korean version of the Brief Encounter Psychosocial Instrument; MET, metabolic equivalent task; HDL, high-density lipoprotein; LDL, low-density lipoprotein; HbA1c, hemoglobin A1C. * < 0.05, ** < 0.01, as compared with baseline values by Wilcoxon signed-rank test.

**Table 3:** Summary of Wilcoxon signed-rank test results.

	Negative Ranks		Positive Ranks		Test Results	
	*n*	Mean Rank	*n*	Mean Rank	Z	*p*-Value
WC (baseline)–WC (3 mo)	18	12.78	4	5.75	–3.368	0.001
WC (baseline)–WC (6 mo)	16	13.38	6	6.50	–2.847	0.004
SBP (baseline)–SBP (3 mo)	14	13.25	7	6.50	–2.435	0.015
DBP (baseline)–DBP (3 mo)	16	11.75	5	8.60	–2.523	0.012
EQ-5D (baseline)–EQ-5D (3 mo)	3	8.67	13	8.46	–2.181	0.029
EQ-5D (baseline)–EQ-5D (6 mo)	3	9.00	13	8.38	–2.125	0.034
EQ VAS (baseline)–EQ VAS (3 mo)	3	7.50	12	8.13	–2.157	0.031
EQ VAS (baseline)–EQ VAS (6 mo)	6	5.00	12	11.75	–2.432	0.015
BDI (baseline)–BDI (3 mo)	15	12.67	6	6.83	–2.594	0.009
BDI (baseline)–BDI (6 mo)	14	12.29	6	6.33	–2.510	0.012
BAI (baseline)–BAI (3 mo)	16	11.25	5	10.20	–2.248	0.025
NKQ (baseline)–NKQ (3 mo)	3	5.00	13	9.31	–2.781	0.005
NKQ (baseline)–NKQ (6 mo)	2	7.00	17	10.35	–3.286	0.001
FG (baseline)–FG (6 mo)	5	5.60	15	12.13	–2.875	0.004
TC (baseline)–TC (6 mo)	5	11.00	17	11.65	–2.322	0.020
HDL-C (baseline)–HDL-C (6 mo)	4	10.88	18	11.64	–2.699	0.007
TG (baseline)–TG (6 mo)	4	6.38	17	12.09	–3.128	0.002
HbA1c (baseline)–HbA1c (6 mo)	3	9.50	18	11.25	–3.041	0.002

WC, waist circumference; SBP, systolic blood pressure; DBP, diastolic blood pressure; EQ-5D, EuroQol 5-dimension scale; EQ VAS, EuroQol visual analogue scale; BDI, Beck Depression Inventory; BAI, Beck Anxiety Inventory; NKQ, Nutritional Knowledge Questionnaire; FG, fasting glucose; TC, total cholesterol; HDL-C, high-density lipoprotein cholesterol; TG, triglyceride; LDL-C, low-density lipoprotein cholesterol; HbA1c, hemoglobin A1C. *p*-value, compared with baseline values by Wilcoxon signed-rank test.

## Data Availability

The data supporting the findings of this study are available on request from the corresponding author. The data are not publicly available due to their containing information that could compromise the privacy of the research participants.
